# Advanced ovarian clear cell carcinoma with RAD50 mutation treated by PARP inhibitor pamiparib combined with anti-angiogenesis therapy: a case report

**DOI:** 10.1097/CAD.0000000000001412

**Published:** 2022-11-15

**Authors:** Xiaoyan Huang, Xiaojian He, Dongliang Li, Xiong Chen, Xi Chen

**Affiliations:** a Department of Medical Oncology, 900TH Hospital of Joint Logistics Support Force, Fujian Medical University; b Affiliated Dongfang Hospital, Xiamen University School of Medicine; c Fujian University of Traditional Chinese Medicine, Fuzhou; d Department of Gastroenterology, 900TH Hospital of Joint Logistics Support Force; e Department of Hepatobiliary Medicine, 900TH Hospital of Joint Logistics Support Force, China

**Keywords:** genetic testing, ovarian clear cell carcinoma, pamiparib, RAD50 mutation, targeted therapy

## Abstract

Ovarian clear cell carcinoma (OCCC) is a relatively uncommon epithelial ovarian malignancy with unique clinical, histopathologic and genetic characteristics. Patients with advanced OCCC have poor outcomes and are resistant to standard chemotherapy. Targeted therapy offers a novel approach for treating OCCC. We report the case of a 45-year-old female patient with advanced OCCC who experienced relapse after standard treatment. Further, a frameshift mutation in the homologous recombination repair-related gene RAD50 (RAD50-p.I371Ffs*8) was identified by genetic testing. Next, the patient had received targeted combination therapy with poly (ADP-ribose) polymerase (PARP) inhibitor pamiparib and bevacizumab, achieving partial remission. Patient’s symptoms improved significantly compared to before. To date, the patient has been followed up for more than half a year with favorable survival and high quality of life. The case report suggested that parmiparib-targeted therapy is a viable treatment option for advanced OCCC patients with RAD50 mutation.

## Introduction

Epithelial ovarian cancer (EOC) has the highest mortality rate among gynecologic malignancies [1]. There are approximately 230 000 new cases and 15 000 deaths occurring annually worldwide. The genomic profile of EOC is markedly heterogeneous. EOC is prone to resistance to standard chemotherapy, leading to a high rate of recurrence [1]. Ovarian clear cell carcinoma (OCCC) was initially thought to originate from mesonephros, and it was not until 1973 that WHO recognized OCCC as a distinct histological subtype of EOC [2,3]. OCCC is a relatively rare epithelial ovarian malignancy with unique clinical, histopathological or genetic features [2,3]. Although the overall prognosis of stage I OCCC is favorable, patients with advanced OCCC have a very poor prognosis, which is associated with resistance to standard chemotherapy [2,3]. Targeted therapies offer novel treatment options for rare tumors, and previous works have reported patients with OCCC can benefit from targeted therapy [4,5]. In this report, we described a patient with recurrent OCCC in whom frameshift mutation in the homologous recombination repair (HRR)-related gene RAD50 (RAD50-p.I371Ffs*8) was identified by genetic testing. After targeted therapy with PARP inhibitor pamiparib, the patient achieved partial remission and clinical symptoms were significantly improved.

## Case report

**Fig. 1:**
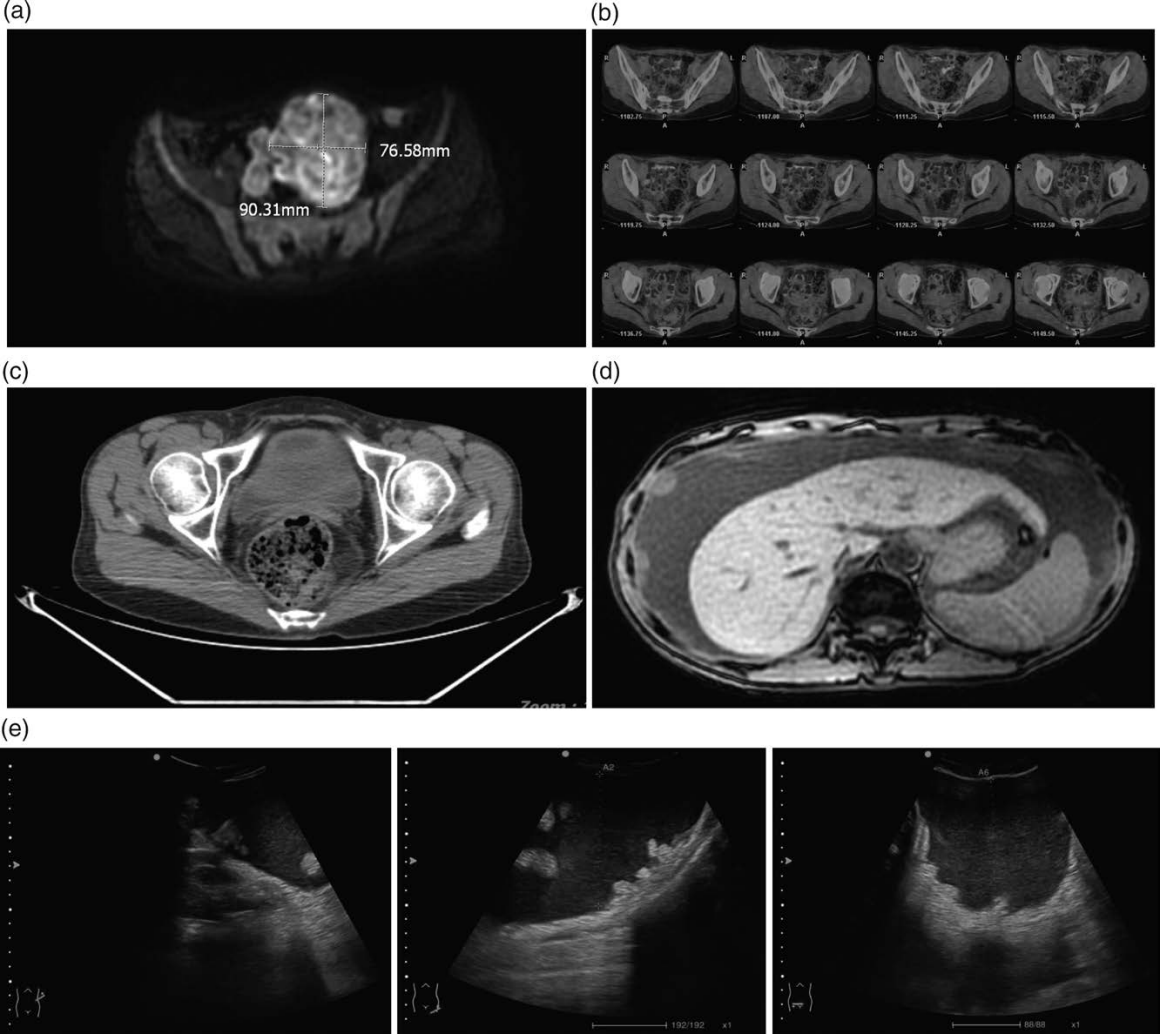
Findings for imaging examinations. (a) Abdominal MRI findings. (b) Abdominal PET-CT. (c) Representative images of CT plain scan. (d) Abdominal CT findings after relapse. (e) Abdominal ultrasound after relapse. CT, computed tomographic.

A 45-year-old female patient who presented with worsening epigastric pain was diagnosed with OCCC on 1 November 2020 (Fig. 1a). The patient who denied any family history was diagnosed with chronic hepatitis B infection 2 years ago and treated with entecavir. Then, the patient underwent abdominal radical surgery for ovarian cancer under general anesthesia on 12 November 2020. Next, standard adjuvant chemotherapy from 23 November 2020 to 14 April 2021 included six cycles of ‘intravenous albumin-bound paclitaxel + intraperitoneal perfusion of cisplatin’ chemotherapy was performed. No sign of tumor recurrence was observed after 3 months (Fig. 1b,c). However, in August 2021, the patient had recurrent abdominal distension without any obvious cause and visited the hospital again. On 13 Aug 2021, an abdominal enhancement MRI indicated the presence of a large amount of ascites, and multiple nodular and mass-like abnormal signals in the abdominal wall, peritoneum, mesentery, liver and spleen, which were considered as tumor metastases (Fig. 1d,e). The renal function of the patient was in the decompensated stage. Renal function parameters were indicated below (urea, 18.7 mmol/L; creatinine, 312.8 μmol/L; uric acid, 696.3 μmol/L; albumin, 28.5 g/L; renal insufficiency, grade 4; physical status score, 2–3). Tumor marker CA125 was significantly elevated. Besides, a routine blood test indicated a low hemoglobin level (66 g/L). Eastern Cooperative Oncology Group (ECOG) performance status was 2.

**Fig. 2:**
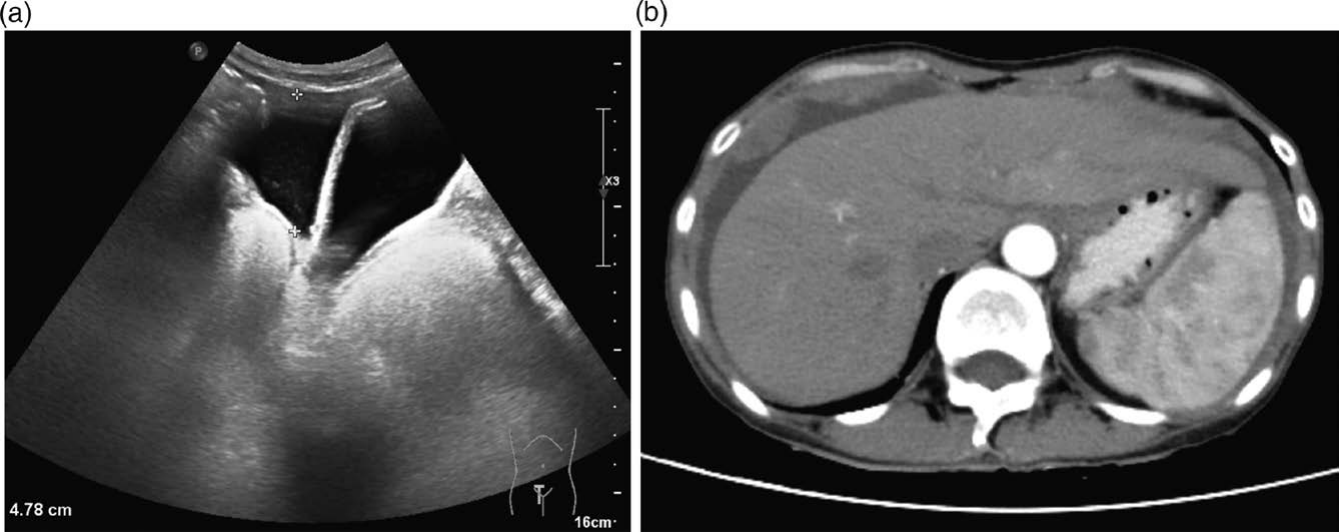
Imaging examinations after targeted therapy in the case. (a) Abdominal ultrasound images. (b) Abdominal CT results. CT, computed tomographic.

**Fig. 3:**
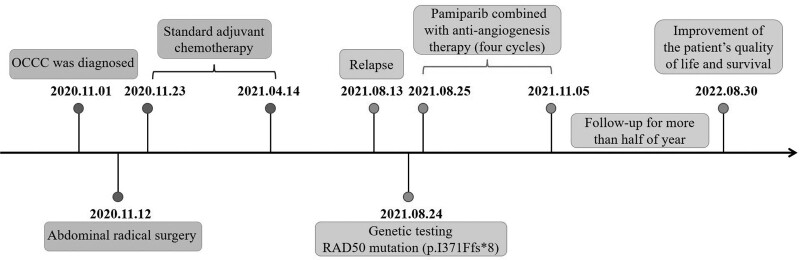
Timeline of disease and treatment.

On 24 August 2021, genetic testing of the ascitic fluid suggested multiple somatic variants, including PIK3CA p.H1047R and CTNNB1 p.d32Y, and variants associated with the HRR-related gene RAD50 (RAD50-p.I371Ffs*8). Tumor mutation burden (TMB) was 0.45 Muts/Mb. The HLA-typing analysis identified a heterozygous mutation in RAD50, indicating better clinical outcomes. Then, on 25 August 2021 the patient was received intraperitoneal perfusion chemotherapy (PRAP inhibitor parmiparib 20 mg po bid + anti-angiogenetic drug bevacizumab 300 mg ivgtt d1/q21d with 5-FU 500 mg d1, d3, d5, d7 + bevacizumab 200 mg d7). After one cycle of treatment, the ascites was significantly reduced (Fig. 2). Renal function indexes were better than before. ECOG performance status was 1. The second to fourth cycles of ‘parmiparib 40 mg po bid + bevacizumab 300 mg ivgtt d1/q21d’ targeted therapy were continued from 21 September 2021 to 5 November 2021. After four cycles of treatment, the patient’s blood routine returned to normal. ECOG performance status was 0. The follow-up was performed until 30 August 2022. This patient had a favorable physical condition (Fig. 3). In summary, PARP inhibitor pamiparib combined with antiangiogenesis therapy helps treat advanced OCCC with extensive peritoneal metastasis and the patient’s quality of life improved significantly.

## Discussion

OCCC is a rare epithelial solid malignancy with unique clinical, histopathological or genetic features. The prognosis of patients with advanced CCCO is very poor; therefore, investigation for effective therapeutic strategies is in urgent demand [2,3]. About 10% of ovarian cancer patients were found to be associated with genetic risk and the first known susceptibility genes are BRCA1 and BRCA2 [6,7]. Other HRR pathway-related genes (ATM, ATR, CDK12, FANCA, FANCD2 and RAD50) were also found to play an essential role in ovarian cancer pathogenesis [8,9]. Due to the limited knowledge about the molecular etiology of ovarian cancer, if only the BRCA1/2 gene is tested, more than 5% of patients with deleterious variants in other known risk genes will be missed [10,11]. A study has suggested that homologous recombination-related genes correlate with the sensitivity of ovarian tumors to radiotherapy and genetic drugs, which could be used as biomarkers for individualized treatment of ovarian tumors [8].

Genetic testing uncovers substantial information about drug targets and helps in precision therapy. RAD50 participates in DNA double-strand break repair, which forms the MRE11-RAD50-NBS1 complex with MRE11 and NBS1. This complex plays an important role in DNA double-strand damage repair, homologous and nonhomologous recombination, checkpoint activation, telomere length maintenance, ensuring smooth DNA replication and maintaining genome stability [12]. The *Rad50* gene was proved to be an important pathogenic mutation in ovarian cancer [13]. In 2020, PARP inhibitors were used for ovarian cancer patients with mutations in 14 HRR genes including RAD50 [14,15], which provides potential therapeutic targets and options for treating ovarian cancer patients with mutations in HRR mutations.

In our case report, the patient with OCCC after recurrence had an ECOG score of 2 and had severely impaired renal function as a result of standard chemotherapy. At this point, the patient was faced with a dilemma; if the patient continually received original chemotherapy, she might not be able to tolerate those side effects. However, if chemotherapy is not administered, survival time may be significantly shortened and she may face death. In the absence of a better solution, our team decided, after rigorous discussions, to perform genetic testing in the hope of finding valuable drug targets. Fortunately, we found the RAD50 mutation, a pathogenic mutation closely related to DNA double-strand break repair. Then, the combination of the RAD50 inhibitor pamiparib with the angiogenesis inhibitor bevacizumab was applied. The patient achieved significant clinical remission and the ECOG score returned to normal. Ascites volume was reduced and tumor volume was diminished. Survival time has exceeded half a year. Our report provides a viable treatment strategy for recurrent OCCC. Genetic testing with targeted drugs brings hope for long-term survival for patients with cancer.

Mutations in the HRR-related gene RAD50 (RAD50-p.I371Ffs*8), a rare mutation in OCCC, resulted in significant relief of clinical symptoms in patients after targeted therapy with the combination of pamiparib and bevacizumab. Overall, genetic testing-based approaches and combined with targeted therapy is a feasible treatment option for advanced or recurrent OCCC.

## Acknowledgements

The authors thank all colleagues in the Department of Oncology, 900TH Hospital of Joint Logistics Support Force, Fujian Province for their support in reporting this case.

The authors received no financial support for the research, authorship, and/or publication of this article.

Conception and design: X.H., X.He., D.L., X.C. and Xi.C. Administrative support: D.L., X.C. and Xi.C. Provision of study materials or patients: X.H., D.L., X.C. and Xi.C. Collection and assembly of data: X.H., X.He. Data analysis and interpretation: X.H., X.He. Manuscript writing: all authors; supervision, X.H., D.L., X.C. and Xi.C. All authors have read and agreed to the published version of the manuscript.

The study was conducted in accordance with the Declaration of Helsinki and approved by the Ethics Committee of 900TH Hospital of Joint Logistics Support Force. Written informed consent was obtained from the patient for publication of this case report and accompanying images.

### Conflicts of interest

There are no conflicts of interest.
